# The Medical Profession and the Public

**Published:** 1869-08-15

**Authors:** John Williamson

**Affiliations:** Chicago


### *The Medical Profession and the Public—A Plea for Popular Professional Education.*

BY JOHN WILLIAMSON, M.D., CHICAGO.

More than any other is the medical profession not in the popular sympathy. The people know nothing of it, and they seem to care less. This is not as it should be, for the profession's sake and the people's. When any one gets sick, without the preparatory knowledge fitting him to discriminate, he sends for what he has learned to call a "doctor." A "good doctor" is to him, not of necessity an educated and refined gentleman, whose versatility of cultivation is his best guarantee of professional excellence, but a man of "good judgment" and "great memory," who remembers a great variety of medicines sure to make a sick person well. There is nothing the public so much demand of the medical practitioner as "experience," and I am quite sure nothing is more perplexing to the unfortunate young physician than this tormenting exaction. To be trustworthy, a "doctor" must have seen very many similarly sick. The intelligent student of medical experience, on the contrary, is perpetually vexed with its conflicting caprice. He would gladly forget his experience. In no other profession is it so unscientific, and, if trusted, so mischievous. Adaptation of intelligent skill to unrelated exigencies is the qualification essential to medical success.

To some "school" of medicine, too, the public always assign the "doctor." To the defense of this "school," the people say, every other consideration is with him subordinate. The people cannot see why schools of medicine are impossible, and the profession will not inform them. In a profession where neither a diagnosis nor a prescription can be repeated, the unity of judgment required to form a "school" is of course forbidden. As well establish conflicting schools of chemistry or divide geometry by the distinction of sect.

So, unfortunately, has the medical education of the masses been neglected by the profession, that I doubt if two per cent. of really intelligent men could tell why one man is a medical "quack," and another a skillful physician. For this state of things the medical profession is altogether responsible. The people do not understand the fundamental canons of medicine. They do not know by what kind of reasoning the profession reaches its conclusions. In consequence of this deficiency of knowledge, medical attention is popularly sought and retained by adventitious partialities. Disease is regarded as an invariable unit, to be combatted by uniform treatment. The people do not see that the philosophy of practical medicine admits of no generalizations, and medical art, in consequence, of no specifics; that reason, rather than memory, is invaluable to guide to successful therapeutics.

The medical profession will never be honored and trusted to an extent commensurate with its merits until the people, its patrons, are by it educated to discriminate familiarly in medical thought. There is no reason why medical men should not instruct the people in medical knowledge. The details of the science must, of course, ever remain with the profession, but the genius of our argument should be understood by all. The people are both honest and intelligent; to be endorsed by them we have but to explain ourselves. No one can see the truth more quickly or embrace it more heartily than the intelligent, though technically uneducated, laity. To say that we can not tell the people what we know, because of the unavoidably scientific conception and expression of our knowledge, is untrue. What one man knows he can communicate to another. Thought, unimpaired by the adaptations of language, is ever communicable. It is not the thought, but the expression, of medical science that repels popular familiarity. With no other class of knowledge are the people more thoroughly interested. An acquaintance with human anatomy and physiology underlies every other successful investigation of the human economy.

No one need fear that to thus instruct the people might eventually dispense with the medical fraternity. The dignity of the profession would be exalted by making those who trust it intelligently critical. It can not be seriously believed that the public safety would thus be endangered. Superficial students of medicine would not then assume the functions of the practitioner, though this affirmative is persistently entertained. Men generally, as we find them in relation to medical knowledge, know just enough, experimentally, to make them annoying and dangerous. With adequate enlightenment the difficulties besetting the best physician are sufficiently known.

Intelligence is always cautious. Of nothing are the ignorant so reckless, and the intelligent more careful, as the human body. Without an acquaintance with primary physiology, from every abuse the restorative powers of medicine seem competent to recover. To the protection of the most cultivated art intelligence always intrusts its most sacred cares. Knowledge sufficient to be critically, but not remedially correct, is what we maintain for the people. "A little knowledge is a dangerous thing," but it is all any of us possess. Opportunities of danger are in proportion just to the revelation which our knowledge makes of them. The amount of one's knowledge is purely relative. The man of great knowledge is the man of great danger.

If we know certain things concerning health and disease, demonstrably, why not tell the people? When the truth is told and proved, no position opposed to it can be maintained. The "quack," then, has no opportunity. The intelligent people will then demand minute conformity to what they have learned as fundamental.

Medical men, in assuming to say that no one is competent to reflect upon the laws of health and disease but the professionally educated physicians, betray a disingenuous pride. There is every reason why the people should thus think. As long as medical men keep profoundly their secrets, the people have no means of distinguishing them from the great body of obvious pretenders. And why should they have? How is the public to decide? What qualifications are they to seek? By what marks may the medical scholar be known? Is he invited to labor in a field where the results of industry and talent are unknown? Must he possess what he can not illustrate, and defend what he can not exhibit? The people say a physician must attest his merits by his successes—he who cures most is most deserving. No criterion could be more fallacious. The people are not competent to award professional success. The veriest charlatans display the most numerous authenticated testimonials of success. Let the primary facts and philosophy of medical science be popularly explained and discussed, and then the merits of a physician may be decided by the measure of his success. The "successful doctor" is now one who possesses the power of convincing his patrons that nature is never a sovereign restorer without his supplementing aid.

To incite popular inquiry here would not be difficult. The injunction "know thyself" is no more obligatory than desirable. What men should do, fortunately they here profoundly feel like doing. Not one conformity to the laws of life, or deviation from them, which they from time to time experience, but what is full of interest to them and easily understood, if divested of a vernacular whose terminology is not absolutely barbarous. The theologian finds no difficulty in securing the attention of the people when the most intangible and abstract propositions are considered. The adjustments of legal questions are of absorbing interest to every one. Why, then, should not medicine, a science profound and faithful, equally merit popular attention? The *knowledge* belonging to both Law and Divinity is *intuitive ;* the *reflection* and *argument* are exegetical and precedented. Knowledge in medicine is eliminated by that exacting co-ordination of logic, induction.

These simple facts the profession should see to it that the people understand.

If the best culture of our profession would write our great and interesting facts, divesting their expression of needless technicality, there is nothing the people would read more readily. Appreciating the demonstrations of our logic, and learning to respect the ardor and honesty of our experimental research, they would exclude from all patronage those swarms of scoundrels who now infest the unwary public.

A few suggestions, to make available what has been said, may be appropriate.

The measure of popular consideration secured by the profession rests with itself. If we are understood, we will be appreciated. Our favorable introduction to the public as men and scholars must come through our now unfortunate patients. They must be intelligently impressed, not only with our individual ability, but with the dignity and absolute truthfulness of our calling. That this impression may be made and may continue, when called to any case of disease not involving the judgment, the practitioner should fully explain the nature of the difficulty and the philosophy of the treatment proposed. To the extent the physician understands the case he can communicate his knowledge to his patient, and, if judiciously, with surpassing profit. In this way he may counteract the ludicrous and often mischievous absurdities of domestic diagnosis and remedy, and, at the same time, secure a profitable review of his own attainments. This fact need not be disguised, that to *appear* competent is as essential as to *be* competent. Appearances, critically construed, are the exclusive means of signifying realities which are fundamental. There is nothing the people like better than reasons assigned and demonstrations exhibited. Medical terms and formula must be eschewed, and the plainest words and reasonings employed. The capability to understand and profit by these explanations must be presupposed by the profession.

To epitomize what I have to say concerning these things, the following summary is offered:

1st. The popular idea that scientific and ordinary knowledge are different, must be corrected.

2d. That public familiarity with the genius and philosophy of medicine would ensure general insecurity.

3d. That adaptation of medical thought and expression to popular taste and comprehension would detract from professional dignity.

4th. The best writers and thinkers in the profession should contribute largely to its popular literature.

5th. A thoroughly appointed periodical literature should be furnished the people and pressed upon their attention by the profession until generally accepted.

6th. The individual practitioner should consider his patient entitled somewhat to his didactic instruction, as he is to his remedial skill.

7th. Books upon systematic medicine, exhaustive and reliable, but popular, should be available to gratify a public taste earnestly longing to be informed.

8th. The popular lecturer in these inviting fields, instead of being as now, an ignoramus or itinerant mountebank, should dispense the best thought of the profession.

9th. General as well as technical scholarship must be exacted of every physician by colleges conferring degrees, or intelligent men of other professions will ridicule our scientific assumptions and gainsay our arguments.

10th. The medical profession must undertake to constitute and exemplify a factor of that great composite, the general public.

